# A scoping review to ascertain the parameters for an evidence synthesis of psychological interventions to improve work and wellbeing outcomes among employees with chronic pain

**DOI:** 10.1080/21642850.2020.1863809

**Published:** 2021-01-28

**Authors:** Joanna L. McParland, Pamela Andrews, Lisa Kidd, Lynn Williams, Paul Flowers

**Affiliations:** a Department of Psychology, School of Health and Life Sciences, Glasgow Caledonian University, Glasgow, Scotland, UK; b School of Medicine, Dentistry and Nursing, University of Glasgow, Glasgow, Scotland, UK; c Department of Psychological Sciences and Health, Faculty of Humanities and Social Sciences, University of Strathclyde, Glasgow, Scotland, UK; d Institute of Health and Wellbeing, University of Glasgow, Glasgow, Scotland, UK

**Keywords:** Scoping review, chronic pain, psychological interventions, employees

## Abstract

Background: Psychological interventions have mixed effects on improving employee outcomes, partly due to significant variability across studies and a lack of focus on mechanisms of action. This scoping review reports on the parameters of these interventions and examines intervention content to bring clarity to this heterogeneous topic area and direct future systematic review work.

Method: Six databases were searched (Cinahl, Cochrane, Embase, Medline, PsychINFO and Web of Science) from April 2010 to August 2020, and a grey literature search was undertaken. Screening was undertaken independently by two authors. The results summarised country, participant and employment characteristics, psychological interventions and work, health and wellbeing outcomes. 10% of the papers were analysed to determine the feasibility of coding intervention descriptions for theory and behaviour change technique (BCT) components.

Results: Database searches yielded 9341 titles, of which 91 studies were included. Most studies were conducted in Europe (78%) and included males and females (95%) ranging in age from 31-56.6 years although other demographic, and employment information was lacking. Musculoskeletal pain was common (87%). Psychological interventions commonly included cognitive behavioural therapy (30%) and education (28%). Most studies employed a randomised control trial design (64%). Over half contained a control group (54%). Interventions were delivered in mostly healthcare settings (72%) by health professionals. Multiple outcomes were often reported, many of which involved measuring sickness absence and return-to-work (62%) and pain and general health (53%). Within the feasibility analysis, most papers met the minimum criteria of containing one paragraph of intervention description, but none explicitly mentioned theory or BCTs.

Conclusion: Psychological interventions for employees with chronic pain vary in their nature and implementation. We have shown scoping reviews can be used to assess the feasibility of applying tools from health psychology to identify the content of these interventions in future systematic review work to improve intervention development.

## Introduction

Pain is a major public health issue that is a burden on health and social care systems across the world. Chronic primary pain, defined as pain in one or more anatomical regions that persists or recurs for longer than three months (Nicholas et al., 2019) affects up to half of the UK population at any one time (Fayaz et al., 2016). Evidence suggests that moderate or severe chronic pain can have a significant adverse impact on an individual’s daily activities, including their working lives (Breivik, Eisenberg, & O’Brien, 2013). Musculoskeletal pain, a common type of chronic pain, is among the leading causes of sickness absence in the UK and Europe and, along with presenteeism and changes in employment status bears a high economic cost associated with reduced productivity in the workplace (Patel et al., 2012; Philips, 2009). This burden is only set to increase with the ageing population and the removal of the retirement age in established economies (Holland & Clayton, 2019). From the employee perspective, not working is problematic because evidence suggests that healthy and safe work confers benefits for physical and mental health, and these benefits outweigh the risks associated with long-term worklessness or prolonged sickness absence (Black, 2008).

To address the burden of chronic pain among employees, there is a clear need for effective interventions to be developed, implemented and scaled-up. Systematic reviews have reported on interventions involving workplace accommodations, service coordination and health service provision including psychological support to reduce sickness absence and increase return-to-work among employees with pain and musculoskeletal disorders, but there is mixed evidence on the effectiveness of these interventions (Finnes et al., 2019; Pike, Hearns, & Williams, 2016; Wainwright, Wainwright, Coghill, Walsh, & Perry, 2019). Some evidence suggests that psychological interventions work best as part of multimodal interventions including e.g. workplace accommodations, and broader psychosocial factors (Cullen et al., 2018; Kamper et al., 2015), however there is little sense of how psychological interventions work or which particular intervention components are associated with effectiveness. One of the key problems with getting useful information out of the published literature is the significant heterogeneity in populations, occupation, content, delivery and setting of interventions (Main & Shaw, 2016).

In the present study, we conducted a scoping review, the first of its kind, to bring clarity to this field through reporting on various parameters within the published literature regarding psychological interventions among employees with chronic pain. The purpose of our scoping review was to shape the direction of future research in the field through identifying gaps in knowledge and defining the parameters for future systematic review work seeking to improve intervention design. The following questions directed the review: (1) What is the volume, geographical scope, population and employment characteristics of psychological interventions for employees with chronic pain?, (2) What are the characteristics of psychological interventions for employees with chronic pain?, (3) What are the primary and secondary outcomes of psychological interventions for employees with chronic pain?

The second aim of our research was to determine the viability of analysing the content of intervention descriptions in this body of literature for theory and behaviour change techniques. We do this by assessing the viability of using tools from health psychology to help resolve problems of heterogeneous intervention descriptions. Within health psychology and behavioural science, a series of tools have recently been developed that provide a common language of intervention components. These are invaluable for resolving problems of varied and inconsistent descriptions of intervention content. From the behaviour change wheel approach (Michie, Strelan, & West, 2011) the common language of intervention content can be thought of on several related levels, including (i) ‘intervention functions’ that consider the type of intervention, (ii) the ‘theoretical domains’, or the causal mechanisms that key intervention components moderate and (iii) the ‘behaviour change techniques’ (BCTs) that are often central to intervention content. Previous work by Palmer et al. (2012) examined BCTs present in interventions for employees with musculoskeletal disorders using an early behaviour change taxonomy but did not examine underlying causal mechanisms and their theoretical relevance. Critically, to be able to use these tools effectively, it must first be established that there are adequate intervention descriptions or manuals available within the published literature. We examined the quality of intervention descriptions within a random sample of papers from the scoping review to determine whether they could usefully be subjected to this type of analysis, allowing us to de-risk this type of analysis in future systematic review work where the analysis of all intervention descriptions would be required.

## Materials and methods

### Approach to the scoping review

A five-stage methodology for scoping reviews was adopted (Arksey & O'Malley, 2005). This involved specifying the research question for the review (stage 1), developing the search strategy for study identification (stage 2), screening and selecting relevant studies (stage 3), developing a data extraction form (stage 4) and collating and summarising the review findings (Stage 5). Details of the stages are provided below. An a priori study protocol guided the conduct of the review. The protocol is unpublished but is available from the first author on request.

### Identification of relevant studies

A comprehensive search strategy was developed in consultation with a specialist subject librarian and experts in the field, including an employer and an employee with pain. Six databases were searched (Cinahl, Cochrane, Embase, Medline, PsychINFO and Web of Science) from 1st April 2010 until 1st April 2019, following the conduct of the last significant review related to this work (Palmer et al., 2012). An updated search was conducted in August 2020. Search terms were searched as keywords and MeSH terms and combined as appropriate. The search strategy was developed and piloted in one database (PsychINFO) before being applied to the remaining databases. The PsychINFO search strategy can be found in supplementary file 1.

Peer-reviewed studies published in English that included adults (>16) with any type of chronic pain, defined as pain for at least three months duration, in paid full or part-time employment, who were either at work or on sick leave were included. Studies examining acute or subacute pain; interventions for employers alone or for those who were unemployed or in voluntary occupations were excluded. Studies involving psychological (including cognitive, behavioural, psychosocial) interventions seeking to improve work and health and wellbeing outcomes among employees with chronic pain were included, while interventions that did not include at least one psychological intervention component and at least one work outcome, or involved the prevention of pain were excluded. Studies could include any design with an intervention component, although editorials, commentaries and case studies were excluded. Any type of control condition could be included and could contain a psychological component if not included in the main intervention. The reference sections of included papers were searched for additional papers, as were the reference sections of relevant book chapters and systematic reviews that were excluded from the scoping review. A limited grey literature search of pain-related and clinical websites and conference abstracts was also undertaken.

### Study selection

Search results were exported into Zotero version 5.0.67.3, where duplicates were removed. Titles and abstracts were initially screened by one author (PA) and following this initial screening process the remaining titles were exported into Covidence. The study inclusion and exclusion criteria were applied to the titles and abstracts which were independently screened by two authors (PA and JM). Independent full-text screening was also undertaken by these authors. Any disagreements between authors were discussed and where consensus could not be reached a third author (LK) was consulted. The update to the review was undertaken by JM and LK. Additionally, screening of reference lists of identified book chapters, included studies and systematic reviews was conducted to identify any potential titles. Finally, conference abstracts and protocols were screened for publication status and possible inclusion.

### Charting, summarising and reporting data

A data extraction tool was developed by the review team and piloted by two authors (PA and LK). One author (PA) independently extracted data from all studies. A second author (JM) independently extracted data from 10% of the studies and checked the extraction undertaken from a further 10% of the studies. Inconsistencies in extraction were resolved through discussion. Information was extracted on the year of study, country of study, number of participants and participate age, gender, ethnicity, education, marital status and pain condition; employment status, occupation and type of organisation (review question 1 (RQ 1)); type of intervention, study design, control condition, participant recruitment, intervention setting and who delivered the intervention (review question 2 (RQ 2)), and primary and secondary outcomes (review question 3 (RQ 3)).

The studies were grouped and categorised by country of origin (Word Health Organisation regions); participant and employment characteristics (age, sex, marital status, pain condition, ethnicity, education, pain condition, employment status, type of occupation and employer); characteristics of the psychological interventions (type of intervention, control condition, study design, recruitment, intervention setting and delivery), and work and health and wellbeing outcomes. The results are presented in the form of a numerical summary of frequencies within the data with an accompanying narrative descriptive summary of the results. We also reported gaps in knowledge that emerged from the narrative synthesis. Data categorisation was undertaken by two authors (PA and PF) and checked by a further two authors (JM and LK).

### Viability of intervention coding

A random sample of n = 9 (10%) of the papers identified within the scoping review were subjected to an analysis of the feasibility of coding intervention descriptions for detailed theoretical content and intervention components to de-risk future work. Two team members (LW and PF) independently examined the presence, length and quality of intervention descriptions, including the presence of explicitly mentioned BCTs using the Behaviour Change Technique Taxonomy v1 (BCTTv1) (Michie et al., 2013), theory of change, and Theoretical Domains Framework (TDF) domains (Cane, O'Connor, & Michie, 2012). We selected these criteria as they provide us with a basis for determining the viability of coding theoretical content and intervention components. We chose to focus on explicitly mentioned BCTs as opposed to those that we could infer to determine the viability of coding these intervention descriptions. If the intervention descriptors require inferred coding this has implications for the time that would be required to undertake this type of analysis in a future systematic review. Studies were examined on whether they (i) provided no intervention description; (ii) provided only a couple of sentences of intervention description; (iii) provided an intervention description that was at least a paragraph (iv) provided an intervention description that was at least and paragraph and contained links to an intervention manual and further resources; (v) provided an intervention description that was at least a paragraph and contained a logic model or theory of change; (vi) provided a description of at least a paragraph and included a logic model, theory of change and made explicit mention of theory/TDF and BCTT plus links to manual/resources. The decision to choose this level of intervention description as a criterion was based on our previous work in this area and reflects that intervention descriptions are typically brief and comprise little detail (De Barra et al., 2018; Hoffman et al., 2014). Accordingly, we determined that having the criterion of a paragraph of intervention description would enable us to determine if there was sufficient information available for theory and BCT coding. We agreed prior to this analysis that at least 50% of the intervention descriptions sampled needed to contain at least one paragraph of intervention description to demonstrate the viability of conducting behavioural analyses in a future systematic review.

## Results

**Figure 1.**
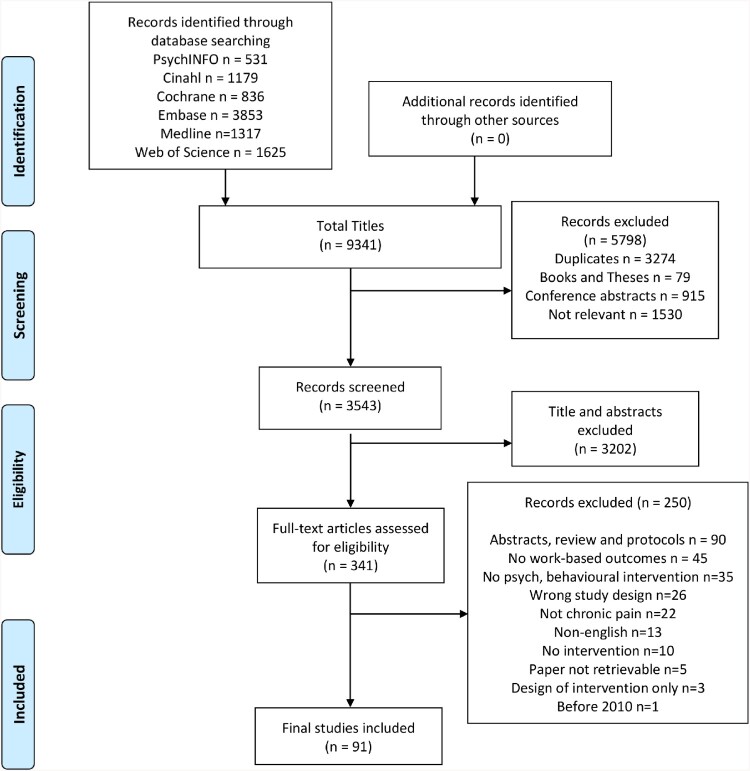
PRISMA flow diagram.

The search of six databases yielded 9341 titles. Following the removal of 3274 duplicates and 2524 papers that failed to meet the a priori inclusion criteria at initial screening, 3543 papers underwent title/abstract screening, with 341 papers undergoing full-text screening. Following this process, 91 papers were included in the final review. A summary of the key features of the included papers is in supplementary file 2. Figure 1 summarises the selection process for the scoping review.

### Country, participant and employment characteristics (RQ 1)

There were 36,108 participants across studies, with sample sizes ranging from 10 to 6709 (mean = 397). With one exception, all studies reported the age characteristics of their sample, with age ranges from 31-56.6 years reported for the whole sample, with the majority of studies reporting a mean age below 50 years (not tabulated). As indicated in Table 1, most studies were conducted in European Countries (78% of studies), particularly Nordic countries. Few studies were conducted in the UK. Most studies included both males and females within the intervention and/or control groups. Over half reported education level, finding participants largely received compulsory education (up to 12 years in primary and secondary education). Fewer studies provided information on marital status or ethnicity. Those that did reported participants to be mostly married and Caucasian. Musculoskeletal pain was reported in most studies, most commonly back/spinal pain and general chronic pain/musculoskeletal pain (Table 1).

**Table 1.** Country, participant and employment characteristics.

Characteristic	Description	Number of studies (% total)
Country		
European country		71 (78)
	Norway	17
	Demark	12
	Sweden	11
	France	6
	Germany	8
	Finland	4
	Switzerland	4
	United Kingdom	3
	Amsterdam	2
	Netherlands	3
	Spain	1
The Americans		15 (17)
	America	10
	Canada	5
West Pacific		4 (4)
	Hong Kong	2
	Australia	1
	Singapore	1
Unclear		1 (1)
Sex	Both males/females included	86 (95)
	One sex reported	4 (4)
	Sex not reported	1 (1)
Pain condition	Back/spinal pain	48 (53)
	Chronic/musculoskeletal	23 (25)
	Neck pain	8 (9)
	Other (e.g. endometriosis)	5 (6)
	Multiple (e.g. mental illness)	4 (4)
	Arthritis	3 (3)
Ethnicity	Not reported	81 (89)
	Reported	10 (11)
Education level	Reported	54 (59)
	Not reported	37 (41)
Marital status	Not reported	65 (71)
	Reported	26 (29)
Type of employer	Not reported	88 (97)
	Reported	3 (3)
Employment status	Not reported	78 (86)
	Reported	13 (14)
Occupation type	Not reported	65 (71)
	Reported	26 (29)
	Blue collar professions	10 (38.5)
	Healthcare/education	10 (38.5)
	White collar professions	5 (19)
	Military	1 (4)

Few studies provided information on type of employer or employment characteristics. Just over a quarter of studies reported the type of job occupied by employees. Among these studies were self-reported ‘blue collar’ occupations (manual labour, cleaning, low skilled) healthcare professions (e.g. nurses, eldercare workers) and ‘white collar’ workers (e.g. management, finance) (Table 1). These findings suggest there are clear gaps in relation to the relatively limited geographical location of the research and in the under-reporting of demographic and employment characteristics.

### Characteristics of the interventions (RQ 2)

The interventions tended to be complex, including multimodal elements for example physiological rehabilitation, exercise or medication management, in addition to psychological components. The psychological components of interventions often contained multiple components that drew explicitly upon approaches such as cognitive behavioural therapy (CBT) (30% of studies) (Angst et al., 2014; Asih, Neblett, Mayer, & Gatchel, 2018; Bergström, Jensen, Hagberg, Busch, & Bergstrom, 2012; Busch, Bodin, Bergstrom, & Jensen, 2011; Campello et al., 2012; Coole, Drummond, & Watson, 2013; Harris et al., 2017; Hartzell, Mayer, & Asih, 2014; Ibrahim, Weber, Courvoisier, & Genevay, 2019; Irvine et al., 2015; Johansen et al., 2019; Jorgensen et al., 2011; Lambeek et al., 2010; Linton et al., 2016; Luthi et al., 2018; Marchand et al., 2015; Mayer, Choi, Howard, & Gatchel, 2013; Mochari-Greenberger, Andreopoulos, Peters, & Pande, 2020; Myhre et al., 2014; Pato et al., 2010; Poulain et al., 2010; Rasmussen et al., 2016; Reme et al., 2016; Sander et al., 2020; Schlicker et al., 2020; Stein & Miclescu, 2013; Vindholmen, Hoigaard, & Haugen, 2016), counselling (8% of studies) (Calner et al., 2017; Ernsen & Lellefjell, 2014; Howard, Mayer, & Gatchel, 2012; Jensen et al., 2012b; Knappe, Briest, & Bethge, 2015; Kold, Hansen, Vedsted-Hansen, & Forman, 2012; Sjöström, Asplund, & Alricsson, 2013), acceptance and commitment therapy (4% of studies) (Berglund et al., 2018; Gismervik et al., 2020; Hara, Bjørngaard, Brage et al., 2018; Hara, Bjørngaard, Jacobsen et al., 2018), motivational interviewing (2% of studies) (Gross et al., 2017; Park et al., 2018) or mindfulness-based stress reduction (1% of studies) (Soler-Font et al., 2019). Other interventions adopted explicit educational approaches (27.5% of studies) (Andersen et al., 2015; Andersen et al., 2016; Bethge, Herbold, Trowitzsch, & Jacobi, 2011; Burton et al., 2016; Busch et al., 2018; Chaléat-Valayer et al., 2016; Tavares Figueiredo et al., 2016; Frederiksen et al., 2017; Hampel, Kopnick, & Roch, 2019; Jensen et al., 2011; Jensen, Jensen, & Nielson, 2012a; Luk et al., 2010; Myhr & Augestad, 2013; Nguyen et al., 2017; Odeen et al., 2013; Pereira et al., 2019; Rantonen et al., 2018; Rantonen et al., 2012; Rantonen et al., 2014; Ree et al., 2016; Saltychev et al., 2014; Sorensen et al., 2010; Stapelfeldt et al., 2011; Streibelt & Bethge, 2014; Werner et al., 2016). These interventions involved psychological strategies e.g. psychoeducation. Other interventions more generally reported the use of psychological and/or behavioural principles (27.5% of studies) (Becker, Angerer, Weber, & Muller, 2020; Beemster, van Bennekom, van Velzen, Frings-Dresen, & Reneman, 2020; Bergstrom, Bergstrom, Hagberg, Bodin, & Jensen, 2010; Bramberg, Bergstrom, Jensen, Hagberg, & Kwak, 2017; Brendbekken et al., 2017; Brendbekken, Vaktskjold, Harris, & Tangen, 2018; Brox et al., 2010; Caby et al., 2016; Hammond et al., 2017; Hampel & Tlach, 2015; Hardison & Roll, 2017; Hartfiel et al., 2017; Hutting et al., 2015; Law et al., 2016; Lebon, Rongières, Apredoaei, & Delclaux, 2017; Lindholdt et al., 2017; McCubbin et al., 2014; Sandsjo et al., 2010; Sullivan & Simon, 2012; Sullivan & Adams, 2010; Sullivan, Adams, & Ellis, 2012; Tan et al., 2016; van Vilsteren et al., 2017a; van Vilsteren et al., 2017b; Westman et al., 2010) (Table 2).

**Table 2.** Intervention, control, design, recruitment and context of interventions.

Variable	Description	Number of studies (% total)
Intervention		
	Cognitive behavioural therapy	27 (30)
	Educational approaches	25 (27.5)
	Interventions containing psychological/behavioural principles	25 (27.5)
	Counselling	7(8)
	Acceptance and commitment therapy	4(4)
	Motivational interviewing	2(2)
	Mindfulness based stress reduction	1(1)
Study design		
	Randomised control trials	58 (64)
	Observational studies (including prospective, retrospective, case series and cohort studies)	31 (34)
	Other	2 (2)
Study part of clinical trial		
	No	60 (66)
	Yes	31 (34)
Control condition		
	Control condition present	49 (54)
	Usual/standard care	30 (61)
	Type of control reported	19 (39)
	No control condition	42 (46)
Method of participant recruitment reported		80 (88)
	Clinical (rheumatology or rehabilitation)	29 (36)
	Health insurance databases	25 (31)
	Employers directly	13 (16)
	GP referral	11 (14)
	Occupational health	2 (3)
Method of participant recruitment not reported		11 (12)
Intervention setting reported		76 (84)
	Number of settings	82
	Outpatient clinic	35 (43)
	Workplace	13 (16)
	Clinical/health centre	13 (16)
	Inpatient setting	11 (13)
	Other (e.g. web based, home)	10 (12)
Intervention setting not reported		15 (16)
Intervention delivery reported		91 (100)
	Number of persons delivering intervention:	217
	Physiotherapist	45 (21)
	General Practitioner/physician/specialist medic	41 (19)
	Psychologist/psychiatrist/counsellor	33 (15)
	Occupational therapist	26 (12)
	Social worker	15 (7)
	Physical/sports therapist	15 (7)
	Nurse	12 (5)
	Other (e.g. hypnotherapist, occupational health)	30 (14)

Most of the studies employed randomised control trial (RCT) designs, including cluster randomised control trials (64% of studies) (Table 2). Thirty-one studies (34%) were part of a registered clinical trial. There was no control condition in n = 42 (46%) of the studies (Angst et al., 2014; Asih et al., 2018; Beemster et al., 2020; Bergstrom et al., 2010; Burton et al., 2016; Caby et al., 2016; Hardison & Roll, 2017; Harris et al., 2017; Hartzell et al., 2014; Ibrahim et al., 2019; Johansen et al., 2019; Lebon et al., 2017; Lindholdt et al., 2017; Luthi et al., 2018; Mayer et al., 2013; McCubbin et al., 2014; Mochari-Greenberger et al., 2020; Pato et al., 2010; Poulain et al., 2010; Reme et al., 2016; Stein & Miclescu, 2013; Vindholmen et al., 2016; Calner et al., 2017; Ernsten & Lillefjell, 2014; Gismervik et al., 2020; Hara, Bjørngaard, Jacobsen et al., 2018; Howard et al., 2012; Jensen et al., 2011; Jensen et al., 2012a; Kold et al., 2012; Luk et al., 2010; Myhr & Augestad, 2013; Pereira et al., 2019; Saltychev et al., 2014; Sorensen et al., 2010; Stapelfeldt et al., 2011; Streibelt & Bethge, 2014; Sjöström et al., 2013; Sullivan & Simon, 2012; Sullivan & Adams, 2010; Sullivan et al., 2012; Tavares Figueiredo et al., 2016). Of the remaining 54% of studies containing a control group, 61% of them ( Berglund et al., 2018; Bergström et al., 2012; Busch et al., 2011; Busch et al., 2018; Campello et al., 2012; Chaléat-Valayer et al., 2016; Frederiksen et al., 2017; Hara, Bjørngaard, Brage et al., 2018; Hartfiel et al., 2017; Hutting et al., 2015; Irvine et al., 2015; Jensen et al., 2012b; Lambeek et al., 2010; Law et al., 2016; Linton et al., 2016; Nguyen et al., 2017; Odeen et al., 2013; Rantonen et al., 2012; Rantonen et al., 2014; Rantonen et al., 2018; Rasmussen et al., 2016; Ree et al., 2016; Sander et al., 2020; Sandsjo et al., 2010; Soler-Font et al., 2019; Tan et al. 2016; van Vilsteren et al., 2017a; van Vilsteren et al., 2017b; Werner et al., 2016; Westman et al., 2010) overtly reported ‘no intervention’ or ‘usual’ or ‘standard’ care, although frequently little detail was provided on what ‘usual care’ consisted of. The remaining studies (39%) reported a specific control condition (Andersen et al., 2015, 2016; Becker et al., 2020; Bethge et al., 2011; Bramberg et al., 2017; Brendbekken et al., 2017; Brendbekken et al., 2018; Brox et al., 2010; Coole et al., 2013; Gross et al., 2017; Hammond et al., 2017; Hampel et al., 2019; Hampel & Tlach, 2015; Jorgensen et al., 2011; Knapp et al. 2015; Marchand et al., 2015; Myhre et al., 2014; Park et al., 2018; Schlicker et al., 2020), for example, education or counselling (Table 2).

Among the studies reporting the method of participant recruitment (88% of studies), the most frequent recruitment method was through a rehabilitation or rheumatology clinic, followed by an individual’s health insurance provider. Interventions were delivered in multiple settings, mostly healthcare settings including inpatient and outpatient clinics and clinical contexts or health centres (Table 2). Interventions were frequently delivered by more than one professional, most frequently physiotherapists, medics, including General Practitioners, psychologists, psychiatrists or counsellors and occupational therapists. Given the complexity of the interventions, it was not possible to determine who delivered the psychological interventions alone, or the setting for the delivery of these interventions. As indicated in Table 2, there was a lack of workplace involvement in the recruitment, setting and delivery of the interventions.

### Outcomes (RQ 3)

It is difficult to definitively report the number of employees were at work or on sick leave at the time of the intervention due to insufficient information or ambiguity around work status reported in some papers. The general pattern indicates that many more employees were on sick leave rather than at work. Over half of the studies (n=53, 58% of studies) did not report secondary outcomes. Forty studies (44 %) reported multiple work-related outcomes (not tabulated).

Table 3 shows the work outcomes measured across studies. More frequently, outcomes addressed sickness absence and return-to-work. Examples of measures of sickness absence and return-to-work include self-reported number of workdays lost due to absence (Andersen et al., 2016; Beemster et al., 2020; Hampel & Tlach, 2015; Law et al., 2016; Poulain et al., 2010; Rasmussen et al., 2016; Jensen et al., 2012a; Gismervik et al., 2020; Knapp et al., 2015; Soler-Font et al., 2019), patient registered data (Bergstrom et al., 2010; Bergström et al., 2012; Brendbekken et al., 2018; Busch et al., 2018; Jensen et al., 2012b; Jorgensen et al., 2011; Odeen et al., 2013; Ree et al., 2016; Reme et al., 2016; Stein & Miclescu, 2013) or disability payments made (Bergstrom et al., 2010; Busch et al., 2011; Gross et al., 2017; Hara, Bjørngaard, Brage et al., 2018; Ibrahim et al., 2019; Jensen et al., 2011; Marchand et al., 2015; Myhre et al., 2014). Other outcomes, some of which were also indicators of return-to-work status, broadly measured the ability to participate in and be productive in work, including self-reported work ability (Table 3). This was measured, for example, using a measure of the Work Ability Index (Becker et al., 2020; Calner et al., 2017; Coole et al., 2013; Frederiksen et al., 2017; Johansen et al., 2019; Jorgensen et al., 2011; Hampel et al., 2019; Knapp et al., 2015; Rasmussen et al., 2016; Saltychev et al., 2014; Sandsjo et al., 2010; Sorensen et al., 2010). A change to employment status and presenteeism were less frequently measured (Table 3).

**Table 3.** Work, health and wellbeing outcomes.

Outcomes	Description	Number of outcomes (% total)
Work outcomes		135
	Sickness absence	46 (34)
	Return to work	38 (28)
	Work ability	28 (21)
	Employment status change (e.g. work type, job status)	14 (10)
	Presenteeism (including productivity)	9 (7)
Health and wellbeing outcomes		228
	Pain	66 (29)
	General health outcomes (e.g. quality of life)	55 (24)
	Mood (depression and anxiety)	43 (19)
	Catastrophising and kinesiophobia	32 (14)
	Function	32 (14)

Health and wellbeing outcomes were measured in most studies (n=81, 89% of studies), with many studies measuring multiple outcomes (n = 75, 82% of studies) (not tabulated). As indicated in Table 3, pain intensity was the most common outcome. This was frequently measured, for example, using the Visual Analogue Scale (Andersen et al., 2015, 2016; Asih et al., 2018; Caby et al., 2016; Chaléat-Valayer et al., 2016; Calner et al., 2017; Coole et al., 2013; Ernsten & Lillefjell, 2014; Gross et al., 2017; Hammond et al., 2017; Hartzell et al., 2014; Jensen et al., 2011; Hutting et al., 2015; Lambeek et al., 2010; Lebon et al., 2017; Luk et al., 2010; Mayer et al., 2013; Myhr & Augestad, 2013; Park et al., 2018; Pato et al., 2010; Rantonen et al., 2012; Rantonen et al., 2014; Rantonen et al., 2018; Sandsjo et al., 2010; Sjöström et al., 2013; Tavares Figueiredo et al., 2016; van Vilsteren et al., 2017a). General health outcomes were also common (Table 3). These were measured, for example, using health-related quality of life measures, such as the SF-36 (Angst et al., 2014; Bethge et al., 2011; Calner et al., 2017; Gross et al., 2017; Howard et al., 2012; Jensen et al., 2011; Jensen et al., 2012b; Knapp et al., 2015; Kold et al., 2012; McCubbin et al., 2014; Mochari-Greenberger et al., 2020; Tan et al., 2016; Westman et al., 2010). Table 3 also indicates that some outcomes were less commonly measured. These outcomes included mood, which was examined using, for example, the Hospital Anxiety and Depression scale (Angst et al., 2014; Berglund et al., 2018; Bethge et al., 2011; Chaléat-Valayer et al., 2016; Coole et al., 2013; Ernsten & Lillefjell., 2014; Hampel & Tlach, 2015; Hara, Bjørngaard, Jacobsen et al., 2018; Harris et al., 2017; Ibrahim et al., 2019; Johansen et al., 2019; Luthi et al., 2018; Marchand et al., 2015; Myhr & Augestad, 2013; Poulain et al., 2010; Reme et al., 2016; Sjöström et al., 2013) catastrophising and kinesiophobia which were frequently measured using the Fear Avoidance and Belief Questionnaire (Campello et al., 2012; Chaléat-Valayer et al., 2016; Coole et al., 2013; Hara, Bjørngaard, Jacobsen et al., 2018; Harris et al., 2017; Jensen et al., 2012b; Marchand et al., 2015; Myhre et al., 2014; Poulain et al., 2010; Tavares Figueiredo et al., 2016; Rantonen et al., 2012; Rantonen et al., 2014; Rantonen et al., 2018; Sorensen et al., 2010) and the Pain Catastrophising Scale (Campello et al., 2012; Hutting et al., 2015; Luthi et al., 2018; Sullivan et al., 2012; Sullivan & Adams, 2012; Sullivan & Simon, 2012; Westman et al., 2010), and physical function that often involved measuring disability using the Oswestry Disability Index (Brox et al., 2010; Campello et al., 2012; Harris et al., 2017; Hartzell et al., 2014; Luk et al., 2010; Marchand et al., 2015; Myhre et al., 2014; Rantonen et al., 2012; Rantonen et al., 2018; Reme et al., 2016; Sander et al., 2020; Schlicker et al., 2020) or the Roland-Morris Disability Questionnaire (Coole et al., 2013; Hartfiel et al., 2017; Lambeek et al., 2010; Jensen et al., 2011; Rantonen et al., 2012; Rantonen et al., 2014; Rantonen et al., 2018; Sorensen et al., 2010; Werner et al., 2016).

### Intervention coding

Table 4 shows that of the 10% sample of papers coded (Bramberg et al., 2017; Gross et al., 2017; Hara, Bjørngaard, Jacobsen et al., 2018; Jensen et al. 2012a; Linton et al., 2016; Sjöström et al., 2013; Sorensen et al., 2010; Tan et al., 2016; van Vilsteren et al., 2017b) the majority met the minimum criteria of containing at least one paragraph of intervention description, with an additional paper also providing a link to further resources. Therefore, as 89% of the sample met the pre-established criteria (50% at Level 2 or better) it can be concluded that it is possible to conduct a meaningful behavioural analysis of the intervention descriptions. In the sample of papers examined we found none that contained a theory of change, logic model, or explicitly mentioned BCTs.

**Table 4.** Coding intervention descriptions.

Level	Detail of intervention description	N	%
Level 0	No intervention description provided	0	0%
Level 1	Only a couple of sentences of intervention description provided	1	11%
Level 2	At least a paragraph on intervention description provided	7	78%
Level 3	Description of at least a paragraph + links to manual/resources	1	11%
Level 4	Description of at least a paragraph + includes a logic model, theory of change	0	0%
Level 5	Description of at least a paragraph + includes a logic model, theory of change + makes explicit mention of theory/TDF and BCTT + links to manual/resources	0	0%

## Discussion

This scoping review, the first of its kind, provides a picture of the heterogeneity within the published literature on psychological interventions for employees with chronic pain. We conducted our review to shape the direction of future research in the field through identifying gaps in knowledge and defining the parameters for future systematic review work. We were also particularly interested in establishing the viability of using tools from health psychology within subsequent systematic reviews to disentangle diverse and inconsistently described interventions.

We found 91 papers reporting interventions that sought to improve work, health and wellbeing outcomes, published between 2010 and 2020. The studies were mostly conducted in European countries, particularly Scandinavian countries. Recipients of the interventions tended to be young to middle-aged employees with a musculoskeletal condition, most commonly back or spinal pain who were recruited through health insurance databases or healthcare settings. There was a high level of complexity within the interventions. Many psychological interventions contained at least some components of CBT and were often delivered as part of multimodal interventions containing complementary non-psychological elements in a RCT. Most of the interventions were delivered by non-psychologists such as physicians and allied health professionals in predominantly healthcare settings. Given the complexity of the interventions, it was not possible to determine who delivered the psychological interventions. Post-intervention outcomes tended to be multi-domain in nature, incorporating various measures concerned with return-to-work and sickness absence, in addition to physical and psychological functioning. The findings of the scoping review are broadly consistent with the pattern of findings reported in recent systematic reviews on interventions for employees with pain and musculoskeletal disorders (Cullen et al., 2018; Finnes et al., 2019; Palmer et al., 2012; Pike et al., 2016; Wainwright et al., 2019). We have contributed to the field through using our broad search strategy to provide detailed clarity on the current state of science in this heterogeneous topic area in a way that is not captured by systematic reviews that often apply specific parameters involving e.g. specific types of psychological interventions or samples, such as those returning to work, to searches. Using our search strategy we were, for example, able to report on a vast array of psychological interventions delivered to employees at work as well as on sick leave at the time of receiving an intervention, although insufficient reporting within papers largely made it difficult to distinguish between both groups.

The scoping review synthesis has identified gaps in knowledge to be addressed in future work. Firstly, more UK-based research is needed as the findings were dominated by research in Scandinavian countries where different welfare systems and employment policies make it difficult to generalise the findings to the UK context that has higher sickness absence rates Holland & Clayton, 2019; Holland & Clayton, 2011). Secondly, given the ageing workforce (Bevan, 2016), psychological interventions for employees over the age of 50 are needed as there was a notable absence of this research within the review. Thirdly, more research is needed to include employers within interventions as evidence suggests that healthcare settings, where most interventions were based, may be insufficient alone for pain management as there is a tendency to focus on clinical outcomes rather than work outcomes (Staal et al., 2013). Fourthly, there was an under-reporting of employment and demographic characteristics across the studies that should be addressed in future research because the decision to return to work is affected not only by medical factors, but also by other, personal factors (Burdoff, 2013; Wilkie & Pransky, 2012). The scoping review findings also lay the foundation for future systematic review work. The review has identified numerous RCTs of psychological interventions that address work, health and wellbeing outcomes among employees with musculoskeletal disorders (Andersen et al., 2015; Andersen et al., 2016; Becker et al., 2020; Berglund et al., 2018; Bergström et al., 2012; Bethge et al., 2011; Busch et al., 2011; Bramberg et al., 2017; Brendbekken et al., 2017; Brendbekken et al., 2018; Brox et al., 2010; Calner et al., 2017; Campello et al. (2012); Chaléat-Valayer et al., 2016; Coole et al. (2013); Hartfiel et al. (2017); Frederiksen et al., 2017; Gismervik et al., 2020; Gross et al., 2017; Hammond et al., 2017; Hampel et al., 2019; Hara, Bjørngaard, Brage et al., 2018; Harris et al., 2017; Hutting et al., 2015; Irvine et al., 2015; Jensen et al., 2011; Jensen et al., 2012a, 2012b; Jorgensen et al., 2011; Knapp et al., 2015; Lambeek et al., 2010; Lindholdt et al., 2017; Linton et al., 2016; Marchand et al., 2015; Mhyre et al., 2014; Nguyen et al., 2017; Odeen et al., 2013; Park et al., 2018; Pato et al., 2010; Pereira et al., 2019; Rasmussen et al., 2016; Rantonen et al., 2012; Rantonen et al., 2014; Rantonen et al., 2018; Ree et al., 2016; Reme et al., 2016; Sander et al., 2020; Sandsjo et al., 2010; Schlicker et al., 2020; Soler-Font et al., 2019; Sorensen et al., 2010; Stapelfeldt et al., 2011; Streibelt & Bethge, 2014; Tan et al., 2016; van Vilsteren et al., 2017a; van Vilsteren et al.. 2017b; Werner et al., 2016; Westman et al., 2010). We have identified a high level of complexity within these interventions that should be addressed in future to disentangle the effects of different intervention components. We have also shown that it is viable to use health psychology tools to analyse intervention descriptions in a field where an analysis of intervention functions, theoretical domains and BCTs is severely lacking within employee interventions. Analysing these interventions in this way may enable within a full systematic review a focussed understanding of the most useful intervention content that could be used in future interventions and address the call for the development of more focussed, theory-led replicable employee interventions that can clearly articulate what works for whom and in which context (Costa-Black, 2014; Main & Shaw, 2020). Since psychological interventions are complex and can be delivered alongside non-psychological interventions, additional interventions e.g. related to workplace accommodations (Main & Shaw, 2020) may also be considered within this evidence synthesis to determine how different types of interventions operate to improve work outcomes.

There are numerous strengths and limitations. One limitation is that only papers published in English were included, and so some key papers may have been missed. Secondly, despite our best efforts we had difficulty accessing the full text for a small number of studies, and so may have excluded relevant papers., Thirdly, while we were able to de-risk future work through determining the viability of coding intervention content for theory and BCTs, in checking only 10% of the papers we did not examine a representative sample of papers. A strength of this research was the use of a comprehensive search strategy which was developed in collaboration with key stakeholders and a subject specialist librarian. Secondly, the review was conducted by an experienced reviewer. Thirdly, the title/abstract and full-text screening was undertaken independently by two authors. Fourthly, we conducted the first test of the viability of using health psychology tools to analyse intervention content in this context.

## Conclusion

There is much variation in the nature and implementation of psychological interventions for employees with chronic pain. The scoping review has provided a picture of the parameters of psychological interventions for employees with chronic pain. We have identified patterns and gaps in knowledge to direct future research. We have also shown that it is possible to use scoping reviews to assess the feasibility of applying tools from health psychology to identify the active content of psychological interventions for employees with pain in future systematic review work to improve intervention development in this heterogeneous field.

## Supplementary Material

Supplemental Material

## Data Availability

Our data extraction table is available from the first author on request.
