# Supplementary material for: A scoping review to ascertain the parameters for an evidence synthesis of psychological interventions to improve work and wellbeing outcomes among employees with chronic pain
Source: Health Psychol Behav Med. 2021 Jan 28;9(1):25–47. doi: 10.1080/21642850.2020.1863809 (PMC8158208; doi:10.1080/21642850.2020.1863809)
Supplement: Supplemental Material [file RHPB_A_1863809_SM3300.zip › Supplementary file 1 sample search strategy.docx]

Supplementary file 1: Search Strategy for PsychINFO

"return to work" OR "return-to-work" OR "sickness absence" OR RTW OR "work* ability" OR "work* productivity" OR "work* readiness" OR "work* loss*" OR "job loss*" OR "work* effectiveness" OR "sick leave" OR sickleave OR absenteeism OR “employee absenteeism” OR presenteeism OR "occupational health" OR "employ* health" OR "sick listed" OR "sick-listed" OR "alternative work" OR "fit note" OR "sick note" OR "re employment" OR "re-employment" OR workability OR "work* disability" OR "work* reintegration" OR reemployment OR "quality of work life" OR "employee efficiency" OR "employee engagement" OR "employee leave benefit*" OR "employee productivity" OR "stay at work" OR "stay-at-work" OR "work* participation" OR "job re-entry" OR "work* engagement" OR "work* performance" OR "occupational health service" OR "job performance"

 Subject headings

“quality of work life” OR reemployment OR “employee absenteeism” OR “employee efficiency” OR “employee engagement” OR “employee leave benefits” OR “employee productivity”

AND

“musculoskeletal pain” OR MSK OR “MSK pain” OR “chronic pain” OR chronic NEAR/3 pain OR “musculoskeletal disorder*” OR “regional pain” OR “locali?ed pain” OR fibromyalgia OR “fibromyalgia syndrome” OR FMS OR “trigeminal neuralgia” OR arthritis OR osteoarthritis OR “rheumatoid arthritis” OR “central neuropathic pain” OR “peripheral neuropathic pain” OR CWP OR “myofascial pain” OR “neuropathic pain” OR “back pain” OR “complex regional pain syndrome” OR CRPS OR “migraine headache” OR pain OR headache OR “nonspecific back pain” OR “non-specific back pain” OR “chronic primary pain syndrome”

Subject headings

“musculoskeletal disorders” OR “myofascial pain” OR “chronic pain” OR arthritis OR “rheumatoid arthritis” OR “neuropathic pain” OR “back pain” OR “complex regional pain syndrome type 1” OR “complex regional pain syndrome type 2” OR “migraine headache” OR headache OR “trigeminal neuralgia” OR pain

AND

“cognitive behavio* therap*” OR CBT OR “behavio* therap*” OR counse?ling OR “cognitive intervention*” OR hypnosis OR hypnotherapy OR “coping strategies intervention*” OR “coping strategies training” OR “mindfulness based stress reduction” OR MBSR OR “mindfulness based cognitive therapy” OR MBCT OR mindfulness OR “acceptance and commitment therapy” OR ACT OR “fear avoidance treatment” OR “motivational interviewing” OR “social support” OR relaxation OR biofeedback OR “diaphragm breathing” OR visuali?ation OR “guided imagery” OR “operant behavio?ral therapy” OR “dialectical behavio?ral therapy” OR “employ* peer support” OR “emotion focu?sed therap*” OR “eye movement desensiti?ation and reprocessing” OR “solution focu?sed brief therap*” OR psychoeducation OR education OR “psycho-education” OR “psycho education” OR self-management OR “self management” OR “progressive muscle relaxation” OR “progressive relaxation therapy” OR “cognitive restructuring” OR “problem solving” OR biopsychosocial OR bio-psychosocial OR “workplace intervention*” OR “employee assistance program*” OR “psychodynamic psychotherapy” OR psychotherapy OR “vocational rehabilitation” OR “social intervention*” OR “vocational intervention*” OR “biopsychosocial approach” OR “bio-psychosocial approach” OR “self help” OR “occupational rehabilitation” OR “coping behavio?r” OR “assertiveness training” OR “social support network” OR imagery OR “imagery psychotherapy” OR “behavio?ral activation” OR “in vivo exposure” OR “self care” OR "self-help " OR "self-care" OR "psychosocial factors" OR “functional restoration (program*)”

Subject headings

“cognitive behavioural therapy” OR “behavioural therapy” OR counselling OR hypnosis OR hypnotherapy OR distraction OR psychotherapy OR “coping behaviour” OR mindfulness OR “acceptance and commitment therapy” OR “assertiveness training” OR “motivational interviewing” OR “social support” OR “social support network” OR “muscle relaxation therapy” OR “progressive relaxation therapy” OR “relaxation therapy” OR “biofeedback training” OR imagery OR “guided imagery” OR “dialectical behaviour therapy” OR “emotion focused therapy” OR “eye movement desensitization training” OR “solution focused therapy” OR “rehabilitation education” OR “psychology education” OR “self-management” OR “workplace intervention” OR “employee assistance programs” OR “psychodynamic psychotherapy” OR “vocational rehabilitation” OR “biopsychosocial approach” OR psychotherapy OR “imagery (psychotherapy)” OR "psychosocial factors"

NOT

Infant* OR children OR child OR adolescent* OR teenager*

>April 2010
