# Supplementary material for: A scoping review to ascertain the parameters for an evidence synthesis of psychological interventions to improve work and wellbeing outcomes among employees with chronic pain
Source: Health Psychol Behav Med. 2021 Jan 28;9(1):25–47. doi: 10.1080/21642850.2020.1863809 (PMC8158208; doi:10.1080/21642850.2020.1863809)
Supplement: Supplemental Material [file RHPB_A_1863809_SM3300.zip › Supplementary file 2 Summary of studies.docx]

| **Author/year** | **Intervention** | **Country** | **Sample size** | **Study design** | **Control**  **Yes/No** | **Outcomes**  **Work/health/wellbeing** |
| --- | --- | --- | --- | --- | --- | --- |
| Berglund et al (2018) | ACT | Sweden | 282 | RCT | Yes | Work, health + wellbeing |
| Gismervick et al (2020) | ACT | Norway | 166 | RCT | No | Work, health + wellbeing |
| Hara et al (2018) | ACT | Norway | 212 | Cohort | No | Work, health + wellbeing |
| Hara et al (2018) | ACT | Norway | 213 | RCT | Yes | Work |
| Angst et al (2014) | CBT | Switzerland | 175 | Cohort | No | Work, health + wellbeing |
| Asih et al (2018) | CBT | USA | 1101 | Cohort | No | Work, health + wellbeing |
| Bergstrom et al (2012) | CBT | Sweden | 194 | RCT | Yes | Work, health + wellbeing |
| Busch et al (2011) | CBT | Sweden | 214 | RCT | Yes | Work |
| Campello et al (2012) | CBT | USA | 33 | RCT | Yes | Work, health + wellbeing |
| Coole et al (2013) | CBT | UK | 51 | RCT | Yes | Work, health + wellbeing |
| Harris et al (2017) | CBT | Norway | 214 | RCT | No | Work, health + wellbeing |
| Hartzell et al (2014) | CBT | USA | 1113 | Retrospective cohort | No | Work, health + wellbeing |
| Ibrahim et al (2019) | CBT | Switzerland | 201 | Prospective Cohort | No | Work, health + wellbeing |
| Irvine et al (2015) | CBT | USA | 597 | RCT | Yes | Work, health + wellbeing |
| Johansen et al (2019) | CBT | Norway | 350 | Non-randomised design | No | Work, health + wellbeing |
| Jorgensen et al (2011) | CBT | Denmark | 294 | RCT | Yes | Work, health + wellbeing |
| Lambeek et al (2010) | CBT | Unclear | 134 | RCT | Yes | Work, health + wellbeing |
| Linton et al (2016) | CBT | Sweden | 140 | RCT | Yes | Work, health + wellbeing |
| Luthi et al (2018) | CBT | Switzerland | 891 | Observational | No | Work, health + wellbeing |
| Marchand et al (2015) | CBT | Norway | 398 | RCT | Yes | Work, health + wellbeing |
| Mayer et al (2013) | CBT | USA | 1631 | Case series | No | Work, health + wellbeing |
| Mochari-Greenberger et al (2020) | CBT | USA | 1086 | Prospective cohort | No | Work, health + wellbeing |
| Myhre et al (2014) | CBT | Norway | 405 | RCT | Yes | Work, health + wellbeing |
| Pato et al (2010) | CBT | Switzerland | 73 | RCT | No | Work, health + wellbeing |
| Poulain et al (2010) | CBT | France | 105 | Prospective cohort | No | Work, health + wellbeing |
| Rasmussen et al (2016) | CBT | Denmark | 594 | RCT | Yes | Work, health + wellbeing |
| Reme et al (2016) | CBT | Norway | 413 | RCT | No | Work, health + wellbeing |
| Sander et al (2020) | CBT | Germany | 295 | RCT | Yes | Work, health + wellbeing |
| Schlicker et al (2020) | CBT | Germany | 76 | RCT | Yes | Work, health + wellbeing |
| Stein & Miclescu (2013) | CBT | Sweden | 51 | Cohort | No | Work, health + wellbeing |
| Vindholmen et al (2016) | CBT | Norway | 74 | Prospective cohort | No | Work, health + wellbeing |
| Calner et al (2017) | Counselling | Sweden | 99 | RCT | No | Work, health + wellbeing |
| Ernsten & Lillefjell (2014) | Counselling | Norway | 92 | Cohort | No | Work, health + wellbeing |
| Howard et al (2012) | Counselling | USA | 2484 | Retrospective cohort | No | Work, health + wellbeing |
| Jensen et al (2012)b | Counselling | Denmark | 224 | RCT | Yes | Work, health and wellbeing |
| Knapp et al (2015) | Counselling | Germany | 307 | RCT | Yes | Work, health + wellbeing |
| Kold et al (2012) | Counselling | Denmark | 10 | Observational pilot | No | Work, health + wellbeing |
| Sjostrom et al (2013) | Counselling | Sweden | 54 | Retrospective | No | Work, health + wellbeing |
| Andersen et al (2016) | Education | Denmark | 141 | RCT | Yes | Work, health + wellbeing |
| Andersen et al (2015) | Education | Denmark | 141 | RCT | Yes | Work, health + wellbeing |
| Bethge et al (2011) | Education | Germany | 236 | RCT | Yes | Work, health + wellbeing |
| Burton et al (2016) | Education | USA | 243 | Cohort | No | Work, health + wellbeing |
| Busch et al (2018) | Education | Sweden | 6709 | Observational | Yes | Work |
| Chaleat-Valayer et al (2016) | Education | France | 342 | RCT | Yes | Work, health + wellbeing |
| Tavares Figueiredo et al (2016) | Education | France | 99 | Retrospective | No | Work, health + wellbeing |
| Frederiksen et al (2017) | Education | Denmark | 495 | RCT | Yes | Work, health + wellbeing |
| Hampel et al (2019) | Education | Germany | 583 | RCT | Yes | Work, health + wellbeing |
| Jensen et al (2011) | Education | Denmark | 351 | RCT | No | Work, health and wellbeing |
| Jensen et al (2012)a | Education | Denmark | 351 | RCT | No | Work |
| Luk et al (2010) | Education | Hong Kong | 65 | Prospective cohort | No | Work, health + wellbeing |
| Myhr & Augestad (2013) | Education | Norway | 191 | Cohort | No | Work, health + wellbeing |
| Nguyen et al (2017) | Education | France | 87 | RCT | Yes | Work, health + wellbeing |
| Odeen et al (2013) | Education | Norway | 1746 | RCT | Yes | Work, health + wellbeing |
| Pereira et al (2019) | Education | Australia | 763 | RCT | No | Work, health + wellbeing |
| Rantonen et al (2018) | Education | Finland | 126 | RCT | Yes | Work, health + wellbeing |
| Rantonen et al (2012) | Education | Finland | 126 | RCT | Yes | Work, health + wellbeing |
| Rantonen et al (2014) | Education | Finland | 181 | RCT | Yes | Work, health + wellbeing |
| Ree et al (2016) | Education | Norway | 857 | RCT | Yes | Work, health + wellbeing |
| Saltychev et al (2014) | Education | Finland | 854 | Prospective cohort | No | Work, health + wellbeing |
| Sorensen et al (2010) | Education | Denmark | 207 | RCT | No | Work, health + wellbeing |
| Stapelfeldt et al (2011) | Education | Denmark | 351 | RCT | No | Work |
| Streibellt & Bethge (2014) | Education | Germany | 102 | RCT | No | Work, health + wellbeing |
| Werner et al (2016) | Education | Norway | 216 | RCT | Yes | Work, health + wellbeing |
| Becker et al ((2020) | Intervention containing psychological/behavioural principles | Germany | 65 | RCT | Yes | Work, health + wellbeing |
| Beemster et al (2020) | Intervention containing psychological/behavioural principles | Netherlands | 470 | Retrospective cohort | No | Work, health + wellbeing |
| Bergstrom et al (2010) | Intervention containing psychological/behavioural principles | Sweden | 146 | Observational | No | Work, health + wellbeing |
| Bramberg et al (2017) | Intervention containing psychological/behavioural principles | Sweden | 159 | RCT | Yes | Work, health + wellbeing |
| Brendbekken et al (2017) | Intervention containing psychological/behavioural principles | Norway | 284 | RCT | Yes | Work |
| Brendbekken et al (2018) | Intervention containing psychological/behavioural principles | Norway | 284 | RCT | Yes | Work, health + wellbeing |
| Brox et al (2010) | Intervention containing psychological/behavioural principles | Norway | 124 | RCT | Yes | Work, health + wellbeing |
| Caby et al (2016) | Intervention containing psychological/behavioural principles | France | 144 | Retrospective | No | Work, health + wellbeing |
| Hammond et al (2017) | Intervention containing psychological/behavioural principles | UK | 55 | RCT | Yes | Work, health + wellbeing |
| Hampel & Tlach (2015) | Intervention containing psychological/behavioural principles | Germany | 84 | Observational (longitudinal) | Yes | Work, health + wellbeing |
| Hardison & Roll (2017) | Intervention containing psychological/behavioural principles | USA | 201 | Cohort | No | Work |
| Hartfiel et al (2017) | Intervention containing psychological/behavioural principles | UK | 151 | RCT | Yes | Work, health + wellbeing |
| Hutting et al (2015) | Intervention containing psychological/behavioural principles | Netherlands | 117 | RCT | Yes | Work, health + wellbeing |
| Law et al (2016) | Intervention containing psychological/behavioural principles | Hong Kong | 245 | Cohort | Yes | Work |
| Lebon et al (2017) | Intervention containing psychological/behavioural principles | France | 20 | Retrospective | No | Work, health + wellbeing |
| Lindholdt et al (2017) | Intervention containing psychological/behavioural principles | Denmark | 160 | RCT | No | Work |
| McCubbin et al (2014) | Intervention containing psychological/behavioural principles | USA | 38 | Prospective cohort | No | Work, health + wellbeing |
| Sandsjo et al (2010) | Intervention containing psychological/behavioural principles | Netherlands & Sweden | 65 | RCT | Yes | Work, health + wellbeing |
| Sullivan & Simon (2012) | Intervention containing psychological/behavioural principles | Canada | 46 | Cohort | No | Work, health + wellbeing |
| Sullivan & Adams (2010) | Intervention containing psychological/behavioural principles | Canada | 48 | Retrospective two cohort | No | Work, health + wellbeing |
| Sullivan et al (2012) | Intervention containing psychological/behavioural principles | Canada | 60 | Feasibility | No | Work, health + wellbeing |
| Tan et al (2016) | Intervention containing psychological/behavioural principles | Singapore | 153 | RCT | Yes | Work, health + wellbeing |
| Van Vilsteren et al (2017)^a^ | Intervention containing psychological/behavioural principles | Amsterdam | 150 | RCT | Yes | Work, health + wellbeing |
| Van Vilsteren et al (2017)^b^ | Intervention containing psychological/behavioural principles | Amsterdam | 150 | RCT | Yes | Work |
| Westman et al (2010) | Intervention containing psychological/behavioural principles | Sweden | 158 | RCT | Yes | Work, health + wellbeing |
| Soler-Font et al (2019) | Mindfulness-based stress reduction | Spain | 473 | RCT | Yes | Work, health + wellbeing |
| Gross et al (2017) | Motivational Interviewing | Canada | 728 | RCT | Yes | Work, health + wellbeing |
| Park et al (2018) | Motivational Interviewing | Canada | 728 | RCT | Yes | Work, health + wellbeing |
